# Watch Out for a Second SNP: Focus on Multi-Nucleotide Variants in Coding Regions and Rescued Stop-Gained

**DOI:** 10.3389/fgene.2021.659287

**Published:** 2021-07-07

**Authors:** Fabien Degalez, Frédéric Jehl, Kévin Muret, Maria Bernard, Frédéric Lecerf, Laetitia Lagoutte, Colette Désert, Frédérique Pitel, Christophe Klopp, Sandrine Lagarrigue

**Affiliations:** ^1^INRAE, INSTITUT AGRO, PEGASE UMR 1348, Saint-Gilles, France; ^2^INRAE, SIGENAE, Genotoul Bioinfo MIAT, Castanet-Tolosan, France; ^3^INRAE, AgroParisTech, Université Paris-Saclay, GABI UMR 1313, Jouy-en-Josas, France; ^4^INRAE, INPT, ENVT, Université de Toulouse, GenPhySE UMR 1388, Castanet-Tolosan, France

**Keywords:** MNV, SNP, variation, rescued stop-gained, *SLC27A4*, FATP4

## Abstract

Most single-nucleotide polymorphisms (SNPs) are located in non-coding regions, but the fraction usually studied is harbored in protein-coding regions because potential impacts on proteins are relatively easy to predict by popular tools such as the Variant Effect Predictor. These tools annotate variants independently without considering the potential effect of grouped or haplotypic variations, often called “multi-nucleotide variants” (MNVs). Here, we used a large RNA-seq dataset to survey MNVs, comprising 382 chicken samples originating from 11 populations analyzed in the companion paper in which 9.5M SNPs— including 3.3M SNPs with reliable genotypes—were detected. We focused our study on in-codon MNVs and evaluate their potential mis-annotation. Using GATK HaplotypeCaller read-based phasing results, we identified 2,965 MNVs observed in at least five individuals located in 1,792 genes. We found 41.1% of them showing a novel impact when compared to the effect of their constituent SNPs analyzed separately. The biggest impact variation flux concerns the originally annotated stop-gained consequences, for which around 95% were rescued; this flux is followed by the missense consequences for which 37% were reannotated with a different amino acid. We then present in more depth the rescued stop-gained MNVs and give an illustration in the *SLC27A4* gene. As previously shown in human datasets, our results in chicken demonstrate the value of haplotype-aware variant annotation, and the interest to consider MNVs in the coding region, particularly when searching for severe functional consequence such as stop-gained variants.

## Introduction

**FIGURE 1:**
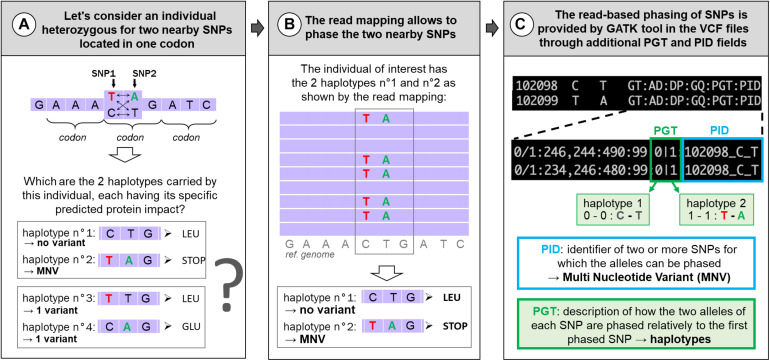
Example of MNVs: predicted impact on the associated protein **(A)** and how to identify them **(B,C)**. **(A)** Example of an MNV composed of two nearby SNPs in one codon and its four potential haplotypes in the population and their predicted impact on the associated protein. In contrast to other haplotypes, haplotype no. 2 contains two variants (T and A) and corresponds to an MNV. **(B)** The IGV (Integrated Genome Browser; Robinson et al., 2011) screen shot indicates the principle of read-based phasing of SNPs: short read mapping against the reference genome of the heterozygous individual allows us to phase both SNPs giving two haplotypes: C with T (reference alleles) on one side and T with A (alternative alleles) on the other side. When translated, these two haplotypes correspond to a leucine or a STOP codon and not to a simple amino acid change (LEU → GLU) if the two haplotypes had been composed by only one reference and one variant as shown in **(A)**. **(C)** Information (PID and PGT) provided by GATK in the VCF files about the phased SNPs according to the read-based phasing shown in **(B)**.

Next-generation sequencing has given access to genomes at the nucleotide level through DNA-seq but also specifically to expressed regions by whole-exome sequencing (WES, originally focusing on exonic parts of the genome) or RNA-seq. These data enable us to call genetic variations by spotting differences between aligned reads and the species reference genome or among aligned reads. Among these genetic variations, single-nucleotide polymorphisms (SNPs) are the most frequent and most studied variations. Although most variations are located in non-coding regions, the most analyzed lie in protein-coding regions where their potential impact(s) on the protein are relatively easy to predict. For example, in a study using 60,706 human exomes, the Exome Aggregation Consortium (ExAC) identified 3,230 genes with near-complete depletion of predicted protein-truncating variants. Of these genes, 72% have not been related to any known human disease phenotype (Lek et al., 2016). Different popular tools have been developed this last decade to predict SNPs’ effects on proteins such as *Variant Effect Predictor* (VEP) (McLaren et al., 2016), SnpEff (Cingolani et al., 2012), or *ANNOtate VARiation* (ANNOVAR) (Wang et al., 2010). But these tools consider each variation location individually, as if it they were specific to “reference” nucleotides. However, SNPs can be grouped by two or more coexisting variants present in the same haplotype (in the same individual), in which case they are called “multi-nucleotide variants” (MNVs). An example of MNV in one individual (with two nearby SNPs) is given in Figure 1. When such MNVs occur within a codon, the amino acid modification caused by this MNV may be different from protein change resulting from each constituent SNPs taken individually, leading to a risk of erroneous functional consequence prediction, as depicted in Figure 1. MNV identification tools have been developed using different methods for phasing SNPs [MAC (Wei et al., 2015), varDic (Lai et al., 2016), COPE (Cheng et al., 2017), BCFtools (Danecek and McCarthy, 2017), and MACARON (Khan et al., 2018)] and have been applied to different human genetic variant datasets [1,000 Genomes Project dataset (Cheng et al., 2017; Danecek and McCarthy, 2017; Khan et al., 2018; Wang et al., 2020), ExAC (Lek et al., 2016), The Cancer Genome Atlas (Lai et al., 2016), or gnomAD consortium (Wang et al., 2020)], mainly based on exomes.

To our knowledge, no study has been conducted on MNVs in livestock species. The aim of this paper is to focus on MNVs occurring in protein-coding regions to provide examples and evaluate the functional consequences of resulting mis-annotations. Considering this aim, we used 9.5M SNPs recently detected in 382 chickens from 767 multi-tissue RNA-seq, enriched by construction in expressed regions and therefore in protein-coding regions. From this 9.5M SNPs, we focused on the 3.3M SNPs with reliable genotypes [see the companion paper (Jehl et al., 2021)]. MNV identification requires properly phased variants, i.e., to be located either on the same haplotype (called therefore MNV) or on two different haplotypes (a case of individual SNPs) (see Figures 1A,B). Different SNP phasing strategies exist: (*i*) population-based phasing, using statistical inference of phase from haplotypes shared among individuals of a large genotyped population; (*ii*) family-based phasing, which analyzes the co-transmission of variants between parents and offsprings; and (*iii*) read-based phasing, which evaluates whether close variants are present on the same reads in the DNA-seq or RNA-seq data. Read-based phasing is particularly relevant for close variants, making this method appropriate for MNV analysis in codons, in which variants fall within a maximum distance of 2 bp from one another. Therefore, in this study, we have chosen to identify MNVs by using the read-based phasing provided by the HaplotypeCaller tool of the GATK toolkit, in the VCF file through additional fields (Figure 1C, with the PID and PGT fields) recently added by the common variant caller (DePristo et al., 2011; Van der Auwera et al., 2013; McKenna et al., 2020).

## Materials and Methods

### SNP Dataset

The 3,276,615 SNPs analyzed in this study have been detected following the method presented in the companion paper (Jehl et al., 2021) using 767 multi-tissue RNA-seq of 382 birds from 11 chicken populations (see Additional File 1). This SNP set corresponds to the union of the SNPs with reliable genotypes found in each population (list available on http://www.fragencode.org/lnchickenatlas.html). Briefly, variant detection was performed for each sample using the HaplotypeCaller tool of GATK toolkit (DePristo et al., 2011; Van der Auwera et al., 2013; McKenna et al., 2020) 3.7.0 with options “--stand_call_conf 20.0,” “--min_base_quality_score 10,” and “--min_mapping_quality_score 20” (which are the defaults values). The “GenotypeGVCFs” function was then used with the option “--stand_call_conf 20.0,” to jointly genotype all these samples into one VCF per tissue. First, biallelic SNPs were then extracted using the “SelectVariant” function with option “--selectType SNP –restrictAllelesTo BIALLELIC.” Variants were filtered using “VariantFiltration” with “QD < 2” and “FS > 30.” Considering genotypes, variants were selected with a “(5.reads.DP) genotype CR ≥ 20%” and a “CR ≥ 50%.” The 11 populations include a red jungle fowl population (RJFh), three broiler populations with one experimental line (FLLL) and two commercial ones (Cobb, HerX), six layer populations with two brown-egg commercial lines (Novo1 and Novo2), two brown-egg experimental populations (RpRm and LSnu), two white-egg or cream-egg experimental populations (FrAg and FAyo), and finally a cross between white- and brown-egg experimental lines (Rmx6).

### Analysis of the Functional Impact of Each Individual SNP in the Coding Regions

VEP v92 (McLaren et al., 2016) with a GTF file enriched in long non-coding genes (“--gtf”) was used for effect prediction of each SNP, with “--everything” and “--total_length” options to respectively, obtain SIFT score predictions and lengths of cDNA, CDS, and protein positions (Ng and Henikoff, 2003; Sim et al., 2012).

### MNV Calling and Recalculation of Consequences

The script to detect the MNV and to calculate the consequences is available in Additional File 2.

### Detection of SNPs Located in the Same Codon

With the information produced by VEP, an ID composed of the “transcriptID” and the “position of the SNP in the coding sequence (expressed in codon number)” was created for each consequence. Through this approach, the same codon of the same transcript supporting at least two different SNPs will have the same ID. Thus, only duplicated IDs were kept as they correspond to those containing two or more SNPs.

#### Detection of Co-located SNPs Carried by the Same Haplotype (MNV)

To test if the SNPs located in the same codon were also present in the same haplotype, the VEP file generated in the previous step and the VCF file were joined by the “SNPid” key, equivalent to “CHR_POS_REF/ALT.” The resulting file (VEP merged to VCF information) contained SNPs on the same codon with additional information about their phase (PID and PGT). Finally, the SNPs which were phased (i.e., same PID) and co-located in a codon were extracted: they correspond to MNVs containing two or three phased SNPs in the same codon.

#### Recalculation of the Consequences

With the R package Biostrings v2.50.2 (Pagès et al., 2021), the associated amino acids were produced for each MNV, and with the same strategy as VEP being adopted, the MNV consequences were established.

### Analysis of MNV Functional Impacts and Comparison With the Constituent SNP Impacts

To compare MNV and independent SNP consequences, we selected only the most impactful consequence per codon for these constituent SNPs using the following order of priority from severe to weak consequences: (1) stop-gained, (2) stop-lost, (3) start-lost, (4) missense_variant, (5) stop-retained_variant, and (6) synonymous_variant.

For MNVs with a missense annotation corresponding to a missense annotation for both constituent SNPs, we distinguished two cases:

– missense MNV with an amino acid different from those predicted by the constituent SNPs (SNP1: Missense A; SNP2: Missense B → MNV: Missense C) and
– missense MNV with an amino acid common to one of two amino acids predicted by the constituent SNPs (SNP1: Missense A; SNP2: Missense B → MNV: Missense A or B).

In order to visualize the results, we produced an alluvial plot using the R “alluvial” package v0.1-2 (Bojanowski, 2020).

### GO or KEGG Term Enrichment Analysis

The enrichment analysis of Kyoto Encyclopedia of Genes and Genomes (KEGG) and Gene Ontology (GO) terms in the gene set of interest was performed using the STRING v11.0. tool (Szklarczyk et al., 2019), and a GO or KEGG term was found significantly enriched if the BH-adjusted *p* ≤ 5%.

### DNA Sequencing of *SLC27A4*

Five microliters of DNA samples was mixed with 5 μl of GoTaq Flexi Buffer 5 ×, 2 μl of MgCl_2_ solution (25 mM), 0.125 μl of GoTaq DNA polymerase (5 U/μl) (Promega, catalog number: M891), 0.5 μl of dNTPs 10 mM, 12.5 μl H_2_O, and 1.25 μl of specific reverse (CATTCCCGTAGTGCCAGAGG) and forward primers (GCACTTTCTGGTGCAAAGCA) at 10 μM. Reaction mixtures were then incubated in a T100 thermal cycler (Bio-Rad, Marne la Coquette, France) for 30 cycles with 30 s at 94°C, 30 s at 60°C, and 30 s at 72°C. The amplification products were then deposited on a 2% agarose gel and sent for sequencing (Genoscreen) to verify their location on the chicken genome.

## Results and Discussion

### Read-Based Phasing for Identification of MNVs

**FIGURE 2:**
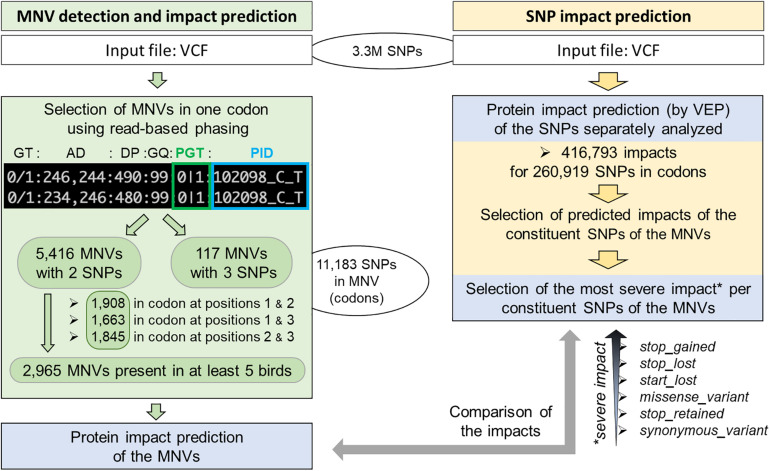
Workflow of MNV detection in coding regions and functional consequence prediction. Left: MNV detection from 3.3M SNPs previously identified using RNA-seq of 382 chickens (companion article; Jehl et al., 2021). Right: MNV constituent SNP selection and protein impact selection of these SNPs separately analyzed by VEP.

Using 3.3M SNPs previously detected from 767 multi-tissue RNA-seq of 382 animals from 11 chicken populations and therefore enriched in coding regions [see the companion paper (Jehl et al., 2021), section “Materials and Methods”], we identified 260,919 unique SNPs in 26,702 transcripts corresponding to 15,835 genes out of 19,545 protein-coding genes (Figure 2, right part—in yellow).

As shown in Figure 2 (left part—in green), we then defined an MNV in a codon as a group of two or three phased SNPs, i.e., existing on the same haplotype in the same individual. We found 11,183 SNPs (4.3% of the SNPs in codons) as constituent variants of 5,533 MNVs, which corresponded to 4,415 transcripts and 2,916 genes. Most of them (98%: 5,416) contained two SNPs with similar proportions (1/3) by constituent SNP position in the codon (1–3, 1–2, and 2–3, Figure 2, left). In order to ensure the reliability of the MNVs, we selected MNVs observed in at least five individuals. Out of the 5,416 MNVs with two SNPs, 2,965 MNVs were present in at least five individuals, corresponding to 2,636 transcripts and 1,792 genes. No GO terms or KEGG terms were found as significantly enriched for this gene list, suggesting that no specific biological pathway was impacted by MNVs. Table 1 gives the distribution of MNVs and their consequences according to the individual number supporting the MNV (ranging from 2 to 100 individuals). We can note that 31% of the 5,416 MNVs with two SNPs are observed only in a single individual and are here considered as erroneous, likely due to sequencing errors.

**TABLE 1:** Occurrences for each type of re-prediction according to the number of individuals carrying the MNV.

SNP annotation	→	MNV annotation	Number of individuals carrying the MNV										
			1	2	3	4	5	10	15	20	30	50	100
	**Eased impact**												
Missense	→	Synonymous	110	91	86	86	**83**	76	74	74	72	66	54
Stop_gained	→	Missense	194	118	102	99	**87**	63	47	39	33	27	14
	**Equal impact**												
Stop_lost	→	Stop_lost	8	4	4	4	**4**	2	2	2	2	1	0
Start_lost	→	Start_lost	19	11	11	11	**11**	8	7	6	5	3	1
Synonymous	→	Synonymous	95	84	81	78	**76**	69	63	58	54	45	32
Missense	→	Missense	4,932	3,425	3,107	2,854	**2,688**	2,162	1,876	1,652	1,369	1,083	716
Stop_gained	→	Stop_gained	12	6	5	5	**4**	3	2	2	2	1	0
	**Aggravated impact**												
Stop_retained	→	Stop_lost	1	0	0	0	**0**	0	0	0	0	0	0
Synonymous	→	Missense	15	6	6	4	**3**	2	1	1	1	1	1
Missense	→	Stop_gained	30	13	11	10	**9**	6	4	3	3	2	1
		**Total MNVs**	5,416	3,758	3,413	3,151	**2,965**	2,391	2,076	1,837	1,541	1,229	819

**FIGURE 3:**
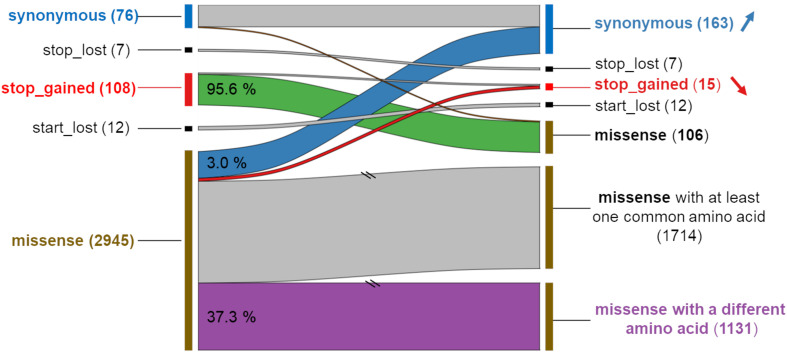
Comparison of the functional impact of MNVs (right) and their component SNPs (left) for each of the 2,965 MNVs. Left: The consequence originally predicted for the component SNPs, the most severe impact being retained by the codon (see Figure 2 or the text for the order). Right: The new prediction associated with the MNVs. For each category of functional predictions of the component SNPs (left), the numbers and percentages are given with new predictions due to the associated MNV. The two slashes indicate that the scale has been adapted (reduction by five times) for better readability.

**The column in bold corresponds to MNVs observed in at least five individuals (MNV set used in Figure 3).**

### Functional Impact Comparison of MNVs and Their Component SNPs

Focusing on the 2,965 MNVs present in at least five individuals, we then compared their functional consequences with those of the 5,930 constituent SNPs as illustrated by the right part of the workflow provided in Figure 2. For such a comparison, we retained for each MNVs, the most severe consequence of the constituent SNPs according to the order indicated in Figure 2 (bottom right). The alluvial plot in Figure 3 depicts the consequence variations before (left) and after (right) taking the MNV impacts into account, according to the different consequence categories; the details of impact variation per MNV are given in Additional File 3 for the whole 2,965 MNV set. We can observe in Figure 3 that the biggest change in variant impacts concerns the originally stop-gained consequence categories, for which 95.6% were re-predicted as missense (green flux: 87 out of the 91 stop-gained initially predicted). The second and third biggest fluxes concern missense consequence categories, for which 37.3% had a different predicted amino acid (violet flux: 1,038 MNVs out of the 2,780 initial missenses), and 3.0% became synonymous variants (blue flux: 83 MNVs out of the initial missenses). The distribution of re-prediction fluxes is provided in Table 1 as a function of the individual number supporting the MNV among the 382 individuals analyzed. Among the 87 rescued stop-gained observed in five individuals, half (47) are observed in at least 15 individuals and are present on average in five populations (see Additional File 4). Out of the MNVs, the proportion of rescued stop-gained MNVs (2.9%), defined as at least one of the individual SNPs creating a nonsense mutation but not the resulting MNV, is in the same order of magnitude as the one reported by the gnomAD consortium with 1,821 rescued stop-gained MNVs out of 31,575 human MNVs (5.8%) (Wang et al., 2020). Genes with a stop-gained MNV rescued in the missense variant are available in Additional File 4 with the population affected and the individual number per population carrying these MNVs. To a lesser extent, nine missenses were re-predicted as stop-gained, which would have gone unnoticed without re-prediction. After a deeper investigation with the IGV browser, these re-predicted stop-gained variants seem to be present since they were not located in a potential exon skipping. Finally, this stop-gained category drastically declined by 86% (from 91 to 13) after considering MNVs, whereas the synonymous category was increased by twofold (from 79 to 159). These different category changes after considering MNVs have a major impact on variant interpretation and thus are critical for accurate variant annotation. More broadly, when the MNVs were considered together, the resulting functional impact differed from the independent impacts of the individual variants in 41.1% of the analyzed MNVs. This large percentage of mis-annotations is relatively consistent with ∼60% of reannotations in human MNVs recently reported by the gnomAD consortium in coding regions (Wang et al., 2020). Such results show the importance of paying attention to these MNVs as highlighted by McLaren et al. (2016): “Current annotation tools, including the VEP, annotate each input variant independently, without considering the potential compound effects of combining alternate alleles across multiple variant loci.”

### Example of an Erroneously Predicted Stop-Gained

**FIGURE 4:**
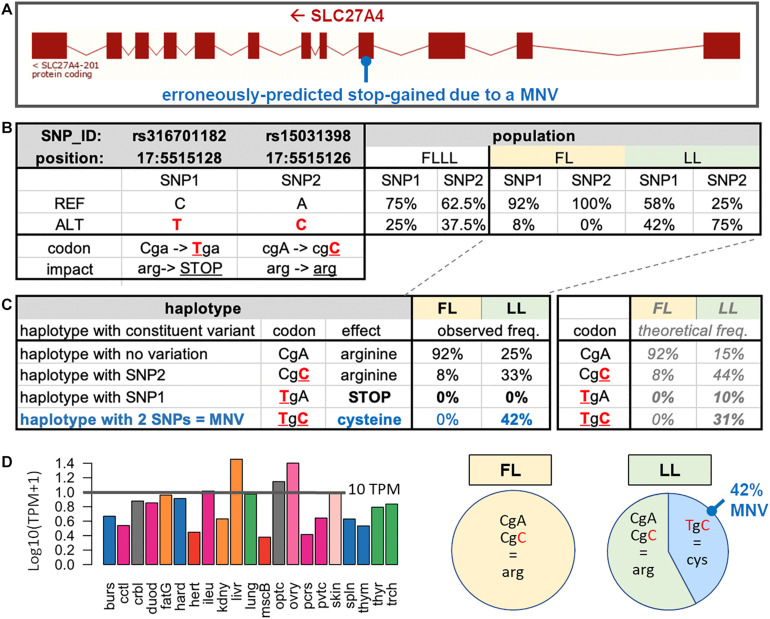
*SLC27A4* with an MNV composed of two phased SNPs observed in the experimental divergent lean line (LL). **(A)** Exon structure of the *SLC27A4* gene and the MNV location. **(B)** For the two SNPs (SNP1: rs316701182 and SNP2: rs15031398) related to the MNV of interest, the allele position (on the Galgal5 genome), functional impact on the associated protein, and frequencies in the FLLL population are indicated, and the two FL and LL subpopulations are divergently selected on abdominal fat weight. **(C)** Effects of the four haplotypes related to SNP1 and SNP2 separately analyzed by VEP and frequencies in LL (*n* = 12) and FL (*n* = 12) subpopulations and focus on the percentage of observed haplotypes in the two FL and LL subpopulations. The haplotypes were determined through the IGV browser of mapped RNA-seq reads against the chicken genome. **(D)** Tissue expression of the gene in a chicken RNA-seq dataset composed of 21 tissues (Jehl et al., 2020).

As an example of erroneously predicted stop-gained, we present the case of the *SLC27A4* gene, which is located on the reverse strand of chicken chromosome 17 (ENSGALG00000004965) (Figure 4A). In this gene, two SNPs rs316701182 and rs15031398, already reported in the Ensembl SNP database (Ensembl, 2018), were respectively, predicted as a stop-gained variant (TGA; stop-gained) and a synonymous variant (CGC; arginine) when compared to the reference haplotype (CGA; arginine) (Figure 4B). These SNPs were present in the FLLL population with frequencies > 20% and interestingly with contrasted frequencies between FL (fat line) and LL (lean line), two subpopulations divergently selected for adipose tissue weight (Leclercq et al., 1980). The rs15031398 SNP is absent in FL (Figure 4B); in the LL population in which we observed both SNPs (Figure 4C), we did not find any animal with the TGA (stop-gained) haplotype (composed of one variant only), with the rs316701182 T variant being always associated with the rs15031398 C variant within the “TGC” MNV. The absence of TGA (stop-gained) haplotype is consistent with several *SLC27A4*-knockout mouse studies which report prenatal lethality (Gimeno et al., 2003) or neonatal lethality (Herrmann et al., 2003; Moulson et al., 2003; Lin et al., 2010; Tao et al., 2012). The *SLC27A4* gene codes fatty acid transport protein 4 (FATP4), which is particularly involved in the uptake of long-chain fatty acids (LCFAs); this gene is highly expressed in various chicken tissues as shown in Figure 4D with an expression > 10 TPM in the liver, ovary, optical system, skin, and intestine (ileum). Interestingly, FATP4 is thought to play a major role in dietary fatty acid uptake in intestinal epithelial cells (Hirsch et al., 1998) and in physiological uptake across cell membranes of LCFAs, which are key metabolites for energy generation and storage; it is viewed as a target to prevent or reverse obesity (Hirsch et al., 1998; Schaffer, 2002). FATP4 could be then related to the lean phenotype of the LL population for two reasons. First, the “TGC” (cysteine) MNV haplotype is reported as a severe change by the SIFT software package compared to the reference “CGA” (arginine) haplotype, suggesting a severe impact on the FATP4 protein function. Second, this “TGC” MNV haplotype is absent in FL birds, whereas it is frequent (42%) in 12 LL birds, with a higher frequency than expected (Figure 4C). We confirmed these results by extending this analysis to 58 birds (29 birds per line) using PCR amplification of the region of interest followed by Sanger sequencing. No rs15031398 was identified in the FL line. In the LL line, we observed 12 birds carrying the “TGC” MNV haplotype (three homozygous and nine heterozygous) and no bird with the TGA (stop-gained) haplotype. These results suggest a strong but not lethal impact of the MNV haplotype on the FATP4 protein function, which could then participate to the lean phenotype of the LL line. However, a genetic association study is needed to support a potential causal link between the FATP4 dysfunctional MNV and a low adiposity in the LL line compared to the FL line.

## Conclusion

We have shown that MNVs represent an important class of genetic variations since they have a significant impact on polymorphism functional interpretation with roughly 40% of MNVs in our dataset inducing reannotation. These reannotations show a decreased impact severity of MNVs when compared to their constituent SNPs, at least for the stop-gained category. As previously demonstrated in human studies, our results in chicken demonstrate the value of haplotype-aware variant annotation and the interest to consider MNVs in coding region particularly when focusing on severe functional consequences such as stop-gained. We illustrated such a case with an erroneous stop-gained annotation found in the chicken *SLC27A4* gene.

## Data Availability Statement

The datasets presented in this study can be found in online repositories. The names of the repository/repositories and accession number(s) can be found in the article/Supplementary Material.

## Author Contributions

FD, FJ, and SL conceived the study and drafted the manuscript. FD, FJ, FL, CK, and SL participated to in bioinformatics processing of the RNA-seq data and bioinformatic analyses. LL, CD, and KM participated in the IGV analysis, PCR amplification, and Sanger sequencing for *SLC27A4* analyses. KM, FP, FL, and CK helped to improve the manuscript. All authors read and approved the final version.

## Conflict of Interest

The authors declare that the research was conducted in the absence of any commercial or financial relationships that could be construed as a potential conflict of interest.

## Supplementary Material

Additional File 1 Detail per population of the number of birds, tissues, and samples. The median number of sequenced fragments (× 1E6) is also indicated for each population.

Additional File 2 Script used for recalculating the consequences for MNVs made up of two SNPs. The script is not generalized and therefore requires adaptations from the users. It is written in bash and R; the change of language is clearly indicated.

Additional File 3 List of MNVs with two phased SNPs (position on the Galgal5 chicken genome) identified among the 382 chickens from 11 populations with their functional impacts (newConseq) and their constituent SNPs (oldConseq). **(A)** List of 2,965 MNVs identified in at least five individuals. **(B)** List of 5,416 MNVs without filtering on the number of individuals supporting the MNV. For the two tables, the population number (popNb), the total number of individuals (indNb_total), and the population name with the individual number per population (popName_indNb), in which the MNVs have been observed, are provided. AA, amino acid.

Additional File 4 List of genes presenting a rescued stop-gained according to the number of individuals carrying the MNV. **(A)** Summary of the number of genes detected as “rescued” according to the number of individuals supporting the MNV. **(B)** For each gene, the population number (popNb) and the population name(s) with the individual number per population (popName:indNb) where the MNV is observed are indicated. The column in blue corresponds to MNVs observed in at least five individuals, the MNV set used in Figure 3.
