# Supplementary material for: Watch Out for a Second SNP: Focus on Multi-Nucleotide Variants in Coding Regions and Rescued Stop-Gained
Source: Front Genet. 2021 Jul 7;12:659287. doi: 10.3389/fgene.2021.659287 (PMC8293744; doi:10.3389/fgene.2021.659287)
Supplement: Additional File 2 — Script used for recalculating the consequences for MNVs made up of two SNPs. The script is not generalized and therefore requires adaptations from the users. It is written in bash and R; the change of language is clearly indicated. [file Table_2.DOCX]

##################################################################################

# #

**#SCRIPT : Detection of MNVs using VCF file & VEP**

# #

##################################################################################

**#### /!\ /!\ /!\ /!\ /!\ /!\ /!\ /!\ ####**

**#### Bash Script ####**

**#### /!\ /!\ /!\ /!\ /!\ /!\ /!\ /!\ ####**

### Files

# General

VCF="[VCF file .gz]"

# For VEP

FASTA="[FASTA file]"

GTF="[VCF file]"

cache="[Cache Version]"

species="[Species Name]"

**### 1) Generation of VEP file**

# → To accelerate the analysis, VEP is used in offline mode (--offline) and with a GTF produced in the lab.

# The --everything and --total_length parameters are used to provide access to the SIFT information and the position of the SNP in the cDNA,

# CDS and protein in the format: "Position/Length".

vep \

--offline \

--species ${species} \

--cache \

--fasta ${FASTA} \

--gtf ${GTF} \

--cache_version ${cache} \

-i ${VCF} \

-o VEP_results.txt \

--everything \

--total_length

VEP="VEP_results.txt"

**### 2) Reduction of the VEP file**

# → Selection of consequences in the coding regions.

grep -v "#" $VEP | grep -P "missense_variant|start_lost|stop_gained|stop_lost|stop_retained_variant|synonymous_variant" > 0b_consSelected.vep

# → Generation of a VEP file of reduced size to facilitate and accelerate the calculations.

# The columns kept are : ($1) Uploaded variation - as chromosome_start_alleles

# ($5) Feature

# ($7) Consequence

# ($10) Position in protein

# ($11) Amino acid change

# ($12) Codon change

# (a) Strand

grep -v "#" 0b_consSelected.vep | awk '{

match($14, /STRAND=-1|STRAND=1/)

a = substr($14,RSTART,RLENGTH)

print $1"\t"$5"\t"$7"\t"$10"\t"$11"\t"$12"\t"a

}' | sed "s/STRAND=//g"> 0c_consSelected_reduced.vep

**### 3) Reduction of the VCF file**

# → List of SNP from the VCF file with the same format as VEP

# i.e. ($1) Uploaded variation - as chromosome_start_alleles

zgrep -v "#" $VCF | awk -F "\t" '{

print $1"_"$2"_"$4"/"$5

}' > 1_VCF_SNPid.list

**#### /!\ /!\ /!\ /!\ /!\ /!\ /!\ /!\ ####**

**#### R Script ####**

**#### /!\ /!\ /!\ /!\ /!\ /!\ /!\ /!\ ####**

**### 4) Extraction of the SNP present by 2 or 3 in a same codon**

## Definition of the working directory

setwd("")

## Imporation of file

# Vep File

VEP <- read.delim("0b_consSelected.vep", dec=".", stringsAsFactors = FALSE, header = FALSE)

colnames(VEP) <- c("Uploaded_variation", "Location", "Allele", "Gene", "Feature", "Feature_type", "Consequence", "cDNA_position", "CDS_position", "Protein_position",

"Amino_acids", "Codons", "Existing_variation", "Extra")

VEP <- VEP[match(unique(VEP$Location), VEP$Location), ]

# VCF File

VCF <- scan("1_VCF_SNPid.list", what = "character")

## Creation of a codon ID [TranscriptID_proteinPosition]

idCodonCreation <- function(x){

return(paste0(x["Feature"], "_", x["Protein_position"]))

}

VEP$codonID <- apply(VEP, 1, idCodonCreation)

## Selection of Codon with MNV

# Only duplicated ID are kept

# supposing the presence of two or three SNPs within the same codon

VEPpop <- VEP[VEP$Uploaded_variation %in% VCF, ]

duplicatedCodon <- as.data.frame(table(VEPpop$codonID))

duplicatedCodon <- duplicatedCodon[duplicatedCodon$Freq >1, ]

print(table(duplicatedCodon$Freq))

res <- VEPpop[VEPpop$codonID %in% duplicatedCodon$Var1, 1]

# List of SNP in MNV

write(res, paste0("1_VCF_MNVid.list"))

**#### /!\ /!\ /!\ /!\ /!\ /!\ /!\ /!\ ####**

**#### Bash Script ####**

**#### /!\ /!\ /!\ /!\ /!\ /!\ /!\ /!\ ####**

**### 5) Extraction of the information contained in the VCF file**

## for each SNP of the previous list

# → Use of the ID format ($1) Uploaded variation - as chromosome_start_alleles

zgrep -v "#" $VCF | awk '{

print $0"\t"$1"_"$2"_"$4"/"$5

}' | grep -f 1_VCF_MNVid.list > 2_VCF_MNVid.vcf

**### 6) Extraction of SNP only phased**

zgrep -v "#" 2_VCF_MNVid.vcf | grep "|" > 3_VCF_MNVid_phased.vcf

**### 7) Extraction of the necessary information : 1) ID / 2) phase**

grep -v "#" 3_VCF_MNVid_phased.vcf | awk -F "\t" '{

for (i = 0; i < NF; i++ ){

match($i, /.\|.:.[^:]*/)

a = substr($i,RSTART,RLENGTH)

array[a] = a

}

printf "%s ",$NF

for (a in array){

printf "%s ",a

}

printf "\n"

delete array

}' | sed "s/1\/0//g" | sed "s/0\/0//g" | sed "s/1\/1//g" | sed "s/0\/1//g" | sed "s/\.\/\.//g" > 4_VCF_MNVid_minimalPhaseInfo.txt

**### 8) Extraction of PID for each SNP**

cat 3_VCF_MNVid_phased.vcf | awk -F '\t' '

BEGIN{

c=0;}

{split($9,test,":");

if (test[6] == "PID") c=6;

if (test[7] == "PID") c=7;

printf "%s ",$NF;

for (i = 10; i < NF; i++ ){

split($i,a,":")

printf "%s ",a[c]}

printf "\n"

}' > 4_idAndPhases.tsv

**### 8) Extraction of the VCF header**

zgrep "#C" $VCF > 4_headerVCF.txt

**### 9) New consequences for MNV with 2 SNP**

**#### /!\ /!\ /!\ /!\ /!\ /!\ /!\ /!\ ####**

**#### R Script ####**

**#### /!\ /!\ /!\ /!\ /!\ /!\ /!\ /!\ ####**

### Definition of the working directory

setwd("")

### Librabries

library(stringr)

library(Biostrings)

### Importing files

# VEP

vep <- read.delim(file = "0c_consSelected_reduced.vep", header = F, sep = "\t", stringsAsFactors = F)

colnames(vep) <- c("id", "transcript", "consequence", "posAA", "AA", "codon", "strand")

# VCF

vcf <- read.delim(file="4_VCF_MNVid_minimalPhaseInfo.txt", header = F, stringsAsFactors = F)

# For annotation

# /!This file depends on the GTF file used. It is to be generated upstream.

# Its structure is as follows :

# gnId tpId

# ENSGALG00000031626 ENSGALT00000065662

# ENSGALG00000031626 ENSGALT00000081419

geneTranscript <- read.delim("", header = T, stringsAsFactors = F)

### Script

## Creation of a dataframe with : 1) ID 2) phased

vcfToDF <- function(x){

idAndphases <- unlist(str_split(x, " "))

idAndphases <- idAndphases[!(idAndphases == "")]

return(c(idAndphases[1], paste0(idAndphases[2:length(idAndphases)], collapse = "$") ))

}

vcf <- data.frame(t(apply(vcf, 1, vcfToDF)), stringsAsFactors = F)

colnames(vcf) <- c("id", "phases")

# Addition of VEP info

res <- merge(vcf, vep, by.x = "id", by.y = "id")

# Creation of an ID (transcript_posAA) which permits to detect MNVd

res$transcriptID <- apply(res[, c("transcript", "posAA")], 1, function(x){return(paste0(x, collapse = "_"))})

transcriptID <- unique(res$transcriptID)

table(table(res$transcriptID))

## Importation of the PID for each SNP

idAndPhases <- read.delim("4_idAndPhases.tsv", header = F, sep = " ", stringsAsFactors = F)

## Treatment of the VCF header

# Parse the header and rename to consider

# individuals and not sample

# ex : RpRm_livr_001 & RpRm_adip_001 → RpRm_001

header <- scan("4_headerVCF.txt", what = "character")

partToKeep <- header[1:9]

partToWork <- header[10:length(header)]

# Depends on the naming, specific to each laboratory

naming <- function(x){

spl <- str_split(x, "_", simplify = T)

pop <- spl[1]

num <- paste0(str_split(spl[3], "\\.", simplify = T)[8:9], collapse = ".")

num <- num[length(num)]

name <- paste0(pop, "_", num)

}

partToWork <- unlist(lapply(partToWork, naming))

# Number of individuals per pop

table(str_sub(unique(partToWork), 1, 4))

# Function to calculate the new consequence considering

# the two SNPs phased

newConsequencesFor2SNP <- function(x){

## Subset of the info corresponding to the IDtranscript

df <- res[res$transcriptID == x, ]

# Test1 : MNV = 2 ?

test1 <- 0

test2 <- 0

if (nrow(df) == 2){test1 <- 1}

## If test OK, we continue

if (test1 == 1){

# Test2 : Are they phased ?

test2 <- 0

# a) Direct link of phased ?

toFind <- str_replace(paste0(unlist(str_split(df[1, "id"], "_"))[2:3], collapse = "_"), "/", "_")

# We eliminate the case 1/0 (Not the same haplotype)

toFind2 <- paste0("0\\|1:", toFind)

toFind3 <- paste0("1\\|1:", toFind)

# Phase with both cases

if (sum(grepl(toFind2, df$phases)) == 2 | sum(grepl(toFind3, df$phases)) == 2){

test2 <- 1

}

if (test2 != 1){

# b) phased by an intermediate ?

# We test if a phase of the first SNP is in the 2 SNP

## SNP1

allPhases <- df[1, "phases"]

# all phases

a1 <- str_split(allPhases, "\\$",simplify = T)

# we delete phases with 1|0 → Not on the same haplotype

b1 <- a1[!grepl("1\\|0", a1, perl = F)]

## SNP2

allPhases <- df[2, "phases"]

# all phases

a2 <- str_split(allPhases, "\\$",simplify = T)

# we delete phases with 1|0 → Not on the same haplotype

b2 <- a2[!grepl("1\\|0", a2, perl = F)]

if (T %in% (b2 %in% b1)) { test2 <- 1}

}

}

## Creation GT ( if 2 SNP and phased)

if (test1 == 1 & test2 == 1) {

#Chrom

chrom <- str_split(df[1, 1], "_", simplify = T)[1]

#Pos ( Default - pos of the first SNP includ in the MNV)

pos <- paste0(lapply(df[, 1 ], function(x){return(str_split(x, "_", simplify = T)[2])}), collapse = ";")

#MNVid

MNVid <- df[1, "transcriptID"]

# Nb of SNP

nbOfSnp <- nrow(df)

# gene

gene <- NA

# transcript

transcript <- str_split(df[1, "transcriptID"], "_", simplify = T)[1]

#Nb of samples

# Consider if the two SNPs are phased for the SAME indivduals

# Extract number of individuals, number of pop and the details

toExtract <- df$id

toAnalyze <- idAndPhases[match(toExtract, idAndPhases$V1), ]

toAnalyze <- rbind(toAnalyze, header)

toAnalyze <- toAnalyze[, -ncol(toAnalyze)]

toAnalyze <- toAnalyze[, (toAnalyze[1, ] != ".") & (toAnalyze[2, ] != ".")]

nbOfSamples <- 0

idIndividuals <- NA

if (is.null(ncol(toAnalyze))){

nbOfSamples <- 0

idIndividuals <- NA

} else if (ncol(toAnalyze) == 2){

if (toAnalyze[1,2] == toAnalyze[2,2]){

nbOfSamples <- 1

idIndividuals <- toAnalyze[3,2]

}

} else {

samples <- apply(toAnalyze[, 2:ncol(toAnalyze)], 2, detectionSample)

samples <- unique(samples)

if (is.na(samples)){

nbOfSamples <- 0

idIndividuals <- NA

} else {

nbOfSamples <- length(samples)

idIndividuals <- paste0(samples, collapse = ":")

}

}

# Codon of the ref seq

codonInitial <- tolower(unlist(str_split(df$codon, "/"))[1])

# Codon of the MNV seq

codonFinal <- NULL

tmp <- unlist(lapply(df$codon, function(x){return(unlist(str_split(x, "/"))[2])}))

for (i in 1:3){

tmp2 <- unlist(lapply(tmp, function(x){return(str_sub(x, i, i))}))

upper <- grep("^[[:upper:]]+$", tmp2, value = T)

if (length(upper) > 0){

codonFinal <- c(codonFinal, upper)

} else {

codonFinal <- c(codonFinal, tmp2[1])

}

}

codonFinal <- paste0(codonFinal, collapse = "")

# AA identificaiton

strand <- df[1,"strand"]

AAinitial <- GENETIC_CODE[toupper(codonInitial)]

AAfinal <- GENETIC_CODE[toupper(codonFinal)]

transcr <- df[1, 'transcriptID']

# Calcul New cosequences

if ((AAinitial == AAfinal) & (AAfinal != "*")){

newCons <-"synonymous_variant"

} else if ((AAinitial == AAfinal) & (AAfinal == "*")) {newCons <-"stop_retained_variant"

} else if ((AAinitial == "*") & (AAfinal != "*")) {newCons <-"stop_lost"

} else if (AAfinal == "*" & AAinitial != "*") { newCons <-"stop_gained"

} else if ((unlist(strsplit(unlist(strsplit(transcr,"_"))[2], "/"))[1] == 1) & AAfinal != "M") {newCons <-"start_lost"

} else if ((unlist(strsplit(unlist(strsplit(transcr,"_"))[2], "/"))[1] == 1) & AAfinal == "M") {newCons <-"start_retained_variant"

} else if ((AAfinal %in% LETTERS) & (AAinitial %in% LETTERS) & (AAfinal != AAinitial)) {newCons <-"missense_variant"

}

# Old consequences

oldCons <- paste0(df$consequence, collapse = "/")

return(c(chrom, pos, MNVid, nbOfSnp, gene, transcript, strand,nbOfSamples, idIndividuals, codonInitial, codonFinal, AAinitial, AAfinal, oldCons, newCons ))

}

}

print("Launch ...")

listFinal2SNP <- pblapply(transcriptID, newConsequencesFor2SNP)

print("End ...")

## Remove Null element

listFinal2SNP <- listFinal2SNP[lengths(listFinal2SNP) != 0]

## MNV recalcul

print("Recalcul ...")

recalculMNV2 <- data.frame(matrix(unlist(listFinal2SNP), nrow=length(listFinal2SNP), byrow=T))

colnames(recalculMNV2) <- c("chrom", "pos", "MNVid", "nbOfSnp", "gene",

"transcript","strand", "nbOfSamples", "idIndividuals", "codonInitial", "codonFinal",

"AAinitial", "AAfinal", "oldCons", "NewCons" )

## Suppression of SNP phase but not in a same sample

recalculMNV2 <- recalculMNV2[recalculMNV2$nbOfSamples != 0, ]

## Match genes transcript

recalculMNV2$gene <- geneTranscript[match(recalculMNV2$transcript, geneTranscript$tpId), "gnId"]

write.table(recalculMNV2, "recalculMNV2.tsv", quote = F, sep= "\t", row.names = F)

**#EOF**
